# Deciphering the shared genetic architecture between schizophrenia and suicide attempt: a statistical genomic study

**DOI:** 10.1186/s12888-026-08189-5

**Published:** 2026-06-03

**Authors:** Bixi Gao, Jiankun Zhou, Bingyi Song, Menghan Wang, Renjie Shou, Youjia Qiu, Juyi Zhang

**Affiliations:** Department of Neurosurgery, The First Affiliated Hospital of Soochow University, Suzhou, Jiangsu Province 215006 China

**Keywords:** Schizophrenia, Suicide attempt, Shared genetic architecture, Genetic correlation, Cross-trait, GWAS

## Abstract

**Background:**

Evidence for reciprocal comorbidity of schizophrenia (SCZ) and suicide attempt (SA) has been discussed in recent years. However, little evidence is known regarding the shared genetic architecture between SCZ and SA. This study investigated the genetic correlation and causality between them.

**Methods:**

Leveraging the genome-wide association summary statistics from European ancestry, we qualified local and global genetic correlations between the two traits through Heritability Estimation from Summary Statistics (*ρ*-HESS) and Linkage Disequilibrium Score Regression (LDSC). Cross-trait meta-analyses, cross-phenotype association, and colocalization analyses were utilized to identify and validate novel risk loci. Bidirectional Mendelian randomization (MR) was used to investigate causality. Tissue-level SNP heritability enrichment was assessed through Stratified LDSC and Multi-marker Analysis of GenoMic Annotation (MAGMA).

**Results:**

LDSC identified significant positive genetic correlation between SCZ and SA (*r*_g_ = 0.48, *p* = 1.23 × 10^− 89^), and *ρ*-HESS observed 13 distinct regions associated with both traits. Cross-trait meta-analysis identified 4,025 significant SNPs across the two phenotypes, corresponding to 79 independent loci after LD-based clumping and removing those in LD region. Then, variant functional annotation identified 44 SNPs as novel risk loci, and colocalization analysis identified five novel shared loci among these SNPs between SCZ and SA, including rs10456045 (PP.H4 97.98%), rs13195401 (PP.H4 93.65%), rs13198474 (PP.H4 95.91%), rs60135207 (PP.H4 89.89%), and rs3132450 (PP.H4 88.68%). In MR analysis, genetically predicted SCZ was positively associated with SA (OR 1.17, 95%CI 1.37 to 1.21, *p* = 7.39 × 10^− 22^), with no reverse causality. Tissue-specific analysis showed that associated genes were predominantly enriched in brain tissues, particularly cortical, limbic, and basal ganglia-related regions.

**Conclusions:**

Our study suggests a shared genetic architecture between SCZ and SA, characterized by overlapping pleiotropic variants, tissue-specific expression, and functionally shared genes. These findings delineate previously underappreciated genetic overlaps between them and provide a framework for future mechanistic exploration and therapeutic target discovery.

**Clinical trial number:**

Not applicable.

**Supplementary Information:**

The online version contains supplementary material available at 10.1186/s12888-026-08189-5.

## Introduction

Schizophrenia (SCZ) encompasses a spectrum of complex psychiatric syndromes characterized by disturbances in perception, thinking, emotion, volitional behavior, and incoherence in mental activity, which typically first happens in adolescence [1, 2]. SCZ causes profound personal suffering and places a heavy psychosocial and economic burden on families and society [3]. Globally, it affects about 1% of individuals and is considered one of the leading 10 causes of disability [4].

**Fig. 1:**
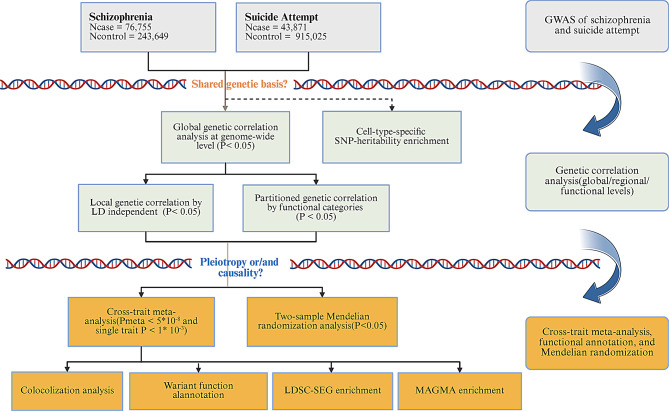
The overview of the study. SNP: Single-nucleotide polymorphism; GWAS: genome-wide association study; LDSC-SEG: Linkage disequilibrium-specific expressed genes; MAGMA: Multi-marker Analysis of GenoMic Annotation

For SCZ, a shorter lifespan is attributed to a higher incidence of suicide behavior. Suicide-related behaviors include suicidal ideation (SI), suicide planning (SP), suicide attempts (SA), and suicide completion (CS) [5]. Unlike thoughts of engagement in suicide and formulations of suicide method, SA refers to non-fatal and self-directed potentially injurious behavior in which there is at least some intent to death [6]. A meta-analysis including 35 studies demonstrated that the pooled lifetime rate of SA was 26.8% in SCZ [7]. Suicide is the most common cause of death in people with a SCZ disorder [8], and young patients are most likely to attempt to kill themselves [9]. Suicide rates are 12 times greater among SCZ patients, and most SA occurs near the onset of disease [10]. Bondy et al. suggested that 43% of the susceptibility of SA could be attributed to genetic association [11]. Fujikane et al. summarized comorbid genetic background of psychiatric disorders in SA, and these correlations persist after accounting for mental disorders [12]. Although epidemiological and clinical studies consistently support a close relationship between SCZ and SA, several important issues remain unexplained. First, it is still unclear to what extent this association reflects a shared genetic architecture, rather than environmental confounding, or reverse causality. Second, while recent large-scale GWAS studies have identified susceptibility loci for SCZ and SA separately [1, 13], and prior studies have shown that SA is genetically correlated with multiple psychiatric phenotypes, the specific shared genetic architecture between SCZ and SA has not been systematically estimated. Third, the biological context between them remains poorly understood, including whether shared signals converge in specific tissues or genomic regions, and whether there exists a causal relationship. Therefore, in the present study, we aimed to comprehensively investigate the genetic overlap between SCZ and SA by quantifying their global and local genetic correlations, identifying shared pleiotropic loci and colocalized genetic variants, characterizing tissue-specific enrichment in both traits, and evaluating potential causal associations using bidirectional Mendelian randomization (MR). By integrating these complementary analyses, we sought to provide a more systematic understanding of the shared genetic architecture between SCZ and SA (Fig. 1).

## Methods

### GWAS summary statistics

We used the GWAS meta-analysis of SCZ that included 90 cohorts of multi-ancestry from the Psychiatric Genomics Consortium (PGC) [14]. This GWAS cohort, involving up to 76,755 patients and 243,649 controls, is reported with the largest sample size of SCZ. The GWAS summary statistics of SA were obtained from the latest GWAS published by Docherty et al., which contained 22 cohorts of multi-ancestry with 43,871 cases and 915,025 controls [15]. Only data of European individuals with high-quality control and without overlapping samples were used. GWAS summary statistic of SCZ of European ancestry (53,386 cases, 77,258 controls, with 7,659,766 SNPs) was acquired from the IEU open GWAS project (ID: ieu-b-5102), which was publicly open access (https://opengwas.io/datasets/), while the European summary data of SA (35,786 cases, 779,392 controls, with 6,861,197 SNPs) was obtained through submitting an application request to the corresponding author. This study was based exclusively on publicly available, de-identified GWAS summary statistics. Ethical approval for the original GWAS datasets was obtained from the corresponding institutional review boards or ethics committees of the contributing cohorts, and informed consent was obtained from all participants. Therefore, no additional ethics approval was required for the present analysis. We filtered out SNPs with minor allele frequency smaller than 1% in the GWAS data and removed SNPs with duplicate or missing identification (rsID) from the data for subsequent analysis. After stringent filtration, 7,632,806 SNPs and 6,861,131 SNPs remained for SCZ and SA, respectively. The locations of all SNPs are referred to the Genome Reference Consortium Human Build 37.

### Genetic correlation and heritability

Linkage disequilibrium score regression (LDSC) was utilized to calculate the heritability and genetic correlations between SCZ and SA using GWAS summary statistics and LD scores of European ancestries from the 1000 Genomes Project [16]. LDSC estimates genetic correlation by regressing test statistics on LD scores. It stays precise even when test statistics are inflated because of polygenicity [17]. Genome-wide genetic correlation (*r*_g_), ranging from − 1 to 1, was used to assess the extent to which two traits share common genetic influences across the genome. A positive *r*_g_ indicates that genetic variants associated with an increased risk of one trait tend to also increase the risk of the other trait, whereas a negative *r*_g_ indicates that genetic variants increasing the risk of one trait tend to decrease the risk of the other trait. Statistical significance was defined as *p* < 0.05.

### Partitioned genetic correlation

We employed Stratified LDSC (S-LDSC) to dissect global genetic correlation, aiming to investigate the contribution of each genomic functional element [18]. This analysis included more than 30 functional elements (including promoter, enhancer, DNase I hypersensitive site, and histone mark-related annotations) that were previously documented in the 1000 Genomes Project Phase 3 reference panel [19]. S-LDSC categorizes SNPs into distinct functional groups, calculates LD scores for each SNP within its assigned category, and uses these scores to evaluate genetic correlations distinct to each functional category [18].

### Tissue-specific enrichment of SNP heritability

#### LDSC

GTEx tissue enrichment analysis was implemented through S-LDSC to identify tissues exhibiting the strongest associations with shared genetic factors. The GTEx V8 dataset comprises 54 tissue types, incorporating SNP variants related to gene expression quantitative traits across diverse tissues. Stratified-LDSC was employed to dissect heritability by leveraging genome-wide SNPs related to SCZ and SA, enabling comparative estimation of trait-specific patterns. Tissue-specific SNP heritability enrichment for both traits was evaluated after adjusting for baseline models and full gene sets, with statistical significance determined via regression coefficient Z-score p-values. Tissue-specific gene expression associations with SCZ and SA were further estimated through targeted analyses. Multiple testing correction was performed using the Bonferroni method, adopting a threshold of *p* < 0.0009 (0.05/54).

#### MAGMA

Tissue-specific and gene-set enrichment analyses using Multi-marker Analysis of GenoMic Annotation (MAGMA) was also conducted as a complement analysis for LDSC [20]. First, gene-level association analyses were carried out using GWAS summary data, estimating the relationship between a gene and the phenotype by averaging the p-values of SNPs near the target gene. Next, gene-set enrichment was carried out with the FUMA set MSigDB_20231Hs_MAGMA; tissue specificity was subsequently evaluated via MAGMA gene-property tests linking tissue-biased expression to disease–gene signals. This analysis was on the basis of data from GTEx V8, which includes expression profiles for 54 tissues.

### Local genetic correlation analysis

Local genetic association was estimated through rho-heritability estimation from summary statistics (*ρ*-HESS) [21]. This algorithm considers 1,702 nearly independent LD intervals with an average span of ~ 1.5 Mb. local SNP heritability for individual traits and their pairwise genetic covariance was estimated using ρ-HESS with the 1,000 Genomes Project EUR reference. These metrics were subsequently integrated to derive region-specific genetic correlation values [21].

### Cross-trait GWAS meta-analysis

We leveraged two cross-trait meta-analytic methods—multi-trait analysis of GWAS (MTAG) and the Cross-Phenotype Association (CPASSOC)—to detect SNPs jointly associated with traits. In MTAG, the SNP effect estimate for trait could be refined by incorporating correlated traits into the analysis [22]. This method increases the probability of detecting loci linked to the studied traits. MTAG operates under the assumptions of uniform SNP heritability across traits and complete genetic covariance between them. To evaluate the validity of this assumption, we calculated the upper bound for the false discovery rate (maxFDR). By leveraging this framework, we improved the detection of shared genetic loci across the traits. CPASSOC was also performed to identify shared risk SNPs [23]. The approach allows for heterogeneous effects among traits and uses a sample-size–weighted meta-analysis of GWAS summary data to obtain SHet and its significant *p*-value. SHet provides more robust statistical power for detecting heterogeneous effects. Using SHet, we combined the summary statistics of SCZ and SA to enhance the analysis [23].

Genome-wide significant SNPs were defined as variants meeting association thresholds in cross-trait meta-analyses (*p* < 5 × 10⁻⁸). GWAS of SCZ and SA were integrated via SHet, followed by clumping in PLINK (v1.9) under the following criteria: primary *p* < 5 × 10⁻⁸, secondary *p* < 1 × 10⁻⁵, LD threshold of r² < 0.2, and physical distance < 500 kb [24]. Independent SNPs were prioritized based on their strongest phenotypic associations. Independent SNPs outside LD with significant cross-trait signals (1-Mb window; r² < 0.2) were classified as novel loci.

### Colocalization analysis

Colocalization analysis through a Bayesian algorithm was carried out to explore the existence of pleiotropic signals colocalized at shared locus, and posterior probability hypothesis four (PP.H4) indicates the locus is linked to both traits with a shared SNP. We classified PP.H4 > 0.8 in favor of the single shared genetic variant between phenotypes [25].

### Bidirectional Mendelian randomization

MR analysis complies with three principles: (1) SNPs were strongly correlated to exposure (relevant assumption); (2) SNPs were not related to confounding factors of the exposure-outcome relationship (independent assumption); (3) SNPs only influence the outcome through exposure (exclusion restriction assumption). Therefore, stringent selection of IVs (*p* < 5 × 10^− 8^) and clumping function parameters were defined with a threshold of r2 < 0.001 and 10,000 kb to secure the independence of each IV and reduce the potential impact of LD [26]. While harmonizing SNP-exposure and SNP-outcome effects, we implemented a filtration step to exclude SNPs with mismatched alleles, palindromic SNPs, and those containing missing values. SNPs associated with potential confounding factors that may influence the causal estimates were removed after a search on the PhenoScanner V2 (http://www.phenoscanner.medschl.cam.ac.uk). Ultimately, IVs with F-statistic > 10 were retained to minimize weak IV bias [27]. The causal links between them were estimated using the inverse variance weighted (IVW) method, supplemented by MR-Egger regression, weighted mode, weighted median, and simple mode. Specifically, IVW was described as the primary and most statistically efficient estimator when the instrumental variable assumptions are satisfied; weighted median and weighted mode were included as more robust alternatives when there were invalid IVs; MR-Egger regression allows directional horizontal pleiotropy through the intercept term, although it is less statistically efficient. Additionally, constrained maximum likelihood-based Mendelian randomization (cML-MA) [28], contamination mixture (ConMix) [29], robust adjusted profile score (MR-RAPS) [30], debiased inverse-variance weighted method (dIVW) [31], Bayesian weighted Mendelian randomization (BWMR) [32], and summary-data-based Mendelian randomization (GSMR) [33] were used as additional robust estimators. cML-MA eliminated the bias caused by related or unrelated pleiotropy, which was more robust than MR-Egger regression. ConMix remained robust when there existed invalid variants with lower mean squared error than weighted mode. MR-RAPS considered special pleiotropy and allowed the existence of weak instruments while providing robust causal estimations. dIVW eliminates bias of weak IVs in primary IVW method and provides robust causal effect under the circumstance of many weak instruments. GSMR is a multi-SNP summary-data-based MR method that estimates causal effects using near-independent instruments and applies the HEIDI-outlier procedure to detect and remove potentially pleiotropic variants [34]. Causal Analysis Using the Summary Effect Estimates (CAUSE) was employed to re-examine significant signals, testing whether the causal model better fit the data than the sharing model based on expected log pointwise posterior density (ΔELPD) [35]. Through analyzing full GWAS results, it could avoid bias induced by sample overlap and horizontal pleiotropy. The outcomes were presented as odds ratios (ORs) and corresponding 95% confidence intervals (CIs). To evaluate the robustness of Mendelian randomization (MR) estimates, sensitivity analyses were systematically performed. To minimize bias due to horizontal pleiotropy, we applied both MR-PRESSO and radial MR to identify and remove outlier SNPs with potentially pleiotropic effects [36]. Cochran’s Q test and I² statistics were employed to quantify heterogeneity across genetic instruments [37]. Scatter plots illustrate trait associations, with regression slopes indicating the magnitude and direction of causal influences. Funnel plots assessed SNP distribution symmetry, where asymmetry suggested potential horizontal pleiotropy. Forest plots displayed causal evaluation derived from individual genetic variants, highlighting result heterogeneity. Leave-one-out analyses iteratively excluded single SNPs to test the influence of individual variants on causal estimates [38].

### MRlap analysis

Considering inevitable and unmeasurable sample overlap in GWAS of European ancestry applied in this study, IVW estimates may be affected, leading to a high number of false positives. We therefore introduced a novel method called MRlap to adjust IVW finding. It assumes the standard MR instrumental variable conditions and models exposure architecture under a spike-and-slab framework, while leveraging cross-trait LDSC to estimate overlap-related bias [39]. When the observed-adjusted effect difference is non-significant (*p* > 0.05), IVW-MR results remain reliable; however, a statistically significant disparity (*p* < 0.05) prioritizes the adjusted effect, which is unaltered by sample overlap. We used a *p*-value threshold of 5 × 10^− 8^, an LD threshold of 0.001, and a distance threshold of 10,000 kb to select instrumental variables in the MRlap analysis.

### Gene-level analysis

GeneMANIA facilitated creation of a gene interaction framework for the overlapping genes. This approach visualized connections between genetic risk-associated genes and their functional neighbors while identifying novel genes with similar biological roles. Additionally, the tool inferred shared gene-related pathways through functional enrichment analysis across multiple gene ontology categories [40]. In addition, spatiotemporal analysis was conducted to map the expression of shared genes across regions of the brain and across development using resources from the Human Brain Transcriptome project (http://hbatlas.org/) [41].

All data analyses were performed using R 4.2.2 software and several associated R packages, including TwoSampleMR (ver.0.56), MendelR (ver 9.2.38), GSMR, MAGMA_Celltyping, MR-PRESSO (ver.1.0), Radial MR, and MRlapPro (ver 1.0.0).

## Results

### Genetic correlation and heritability

A total of 1,131,212 SNPs of SA and 1,217,311 SNPs of SCZ were included for LDSC analysis. The observed-scale heritability was 0.38 and 0.02 for SCZ and SA, respectively. The liability-scale heritability was 0.21 and 0.06 for SCZ and SA considering the prevalence rate (SCZ: 1%; SA: 2%) [15, 42]. Pairwise LDSC showed a positive genome-wide correlation of SA and SCZ (*r*_g_ = 0.48, *p* = 1.2 × 10^− 89^). The result was also confirmed under the constrained intercept condition (*r*_g_ = 0.64, *p* = 1.08 × 10^− 280^) (Table 1).

**Table 1:** Genome-wide genetic correlations between SCZ and SA using constrained and unconstrained LDSC

Method	Trait 1	Observed h^2^_trait_	Liability h^2^_trait_	Trait 2	Observed h^2^_trait_	Liability h^2^_trait_	*r*_g_	*P*-value
LDSC (constrained intercept)	SCZ	0.38	0.21	SA	0.02	0.06	0.48	1.23 × 10^–89^
LDSC (no constrained intercept)		0.41			0.02		0.64	1.08 × 10^− 280^

SCZ: Schizophrenia; SA: Suicide attempts; LDSC: Linkage disequilibrium score regression

### Local genetic correlation

**Fig. 2:**
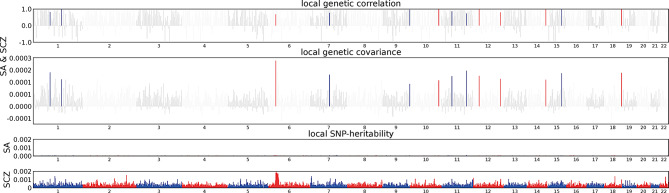
Local genetic correlation and genetic covariance between SCZ and SA, and local SNP heritability estimation for SCZ and SA respectively. SCZ: Schizophrenia; SA: Suicide attempts; SNP: Single-nucleotide polymorphism

The *ρ*-HESS approach was utilized to assess the local genetic correlation between the two traits. Following multiple adjustments, significant local correlations were identified in 13 distinct regions and different chromosomes, led by 12q21.31-21.33 (chr12: 23820634–25371083) (*p* = 6.34 × 10^− 8^), 15q25.2–25.3 (chr15: 80860978–84260468) (*p* = 3.62 × 10^− 7^), and 6p22.1–21.33 (chr6: 25684587–26791233) (*p* = 2.35 × 10^− 7^). (Table S1 and Fig. 2). Notably, all significant regions exhibited positive local estimates, suggesting that the shared genetic effects between the two traits were consistently concordant across these regions.

### Partitioned genetic correlation

SNPs linked to SCZ demonstrated enrichment in 20 of 97 functional categories, with the highest enrichment observed in Human Promoter Villar (Enrichment = 12.71, *p* = 3.58 × 10^− 5^) (Table S2). Similarly, SNPs associated with SCZ were enriched in 10 of 97 functional categories, led by Conserved_Primate (Enrichment = 15.76, *p* = 4.53 × 10^− 7^) (Table S3). Eight functional categories were shared between traits (Table S4). To explore genetic overlaps within these categories, we calculated s-LDSC between them, and significant correlations were observed at Human_Promoter_Villar (*r*_g_ = 0.03) and CpG_Content (*r*_g_ = 0.01) (Table S4).

### Identification of SNPs from cross-trait GWAS meta-analysis

Cross-trait meta-analysis using MTAG and CPASSOC identified genome-wide significant shared association signals between SCZ and SA (Tables S5 and S6). MTAG detected 4,355 significant SNPs, whereas CPASSOC identified 13,681 significant SNPs. To improve robustness of cross-trait results, we focused on signals supported by both methods, yielding 4,025 overlapping SNPs (Table S7). After removing SNPs that were already genome-wide significant in the original SCZ or SA, or those in LD (r² ≥ 0.02) with previously reported loci, 79 novel and independent SNPs remained (Table S8). Afterwards, these SNPs were annotated through VEP and the 3DSNP website, and 44 of them were annotated as novel risk loci for colocalization analysis (Table S9).

### Colocalization

**Fig. 3:**
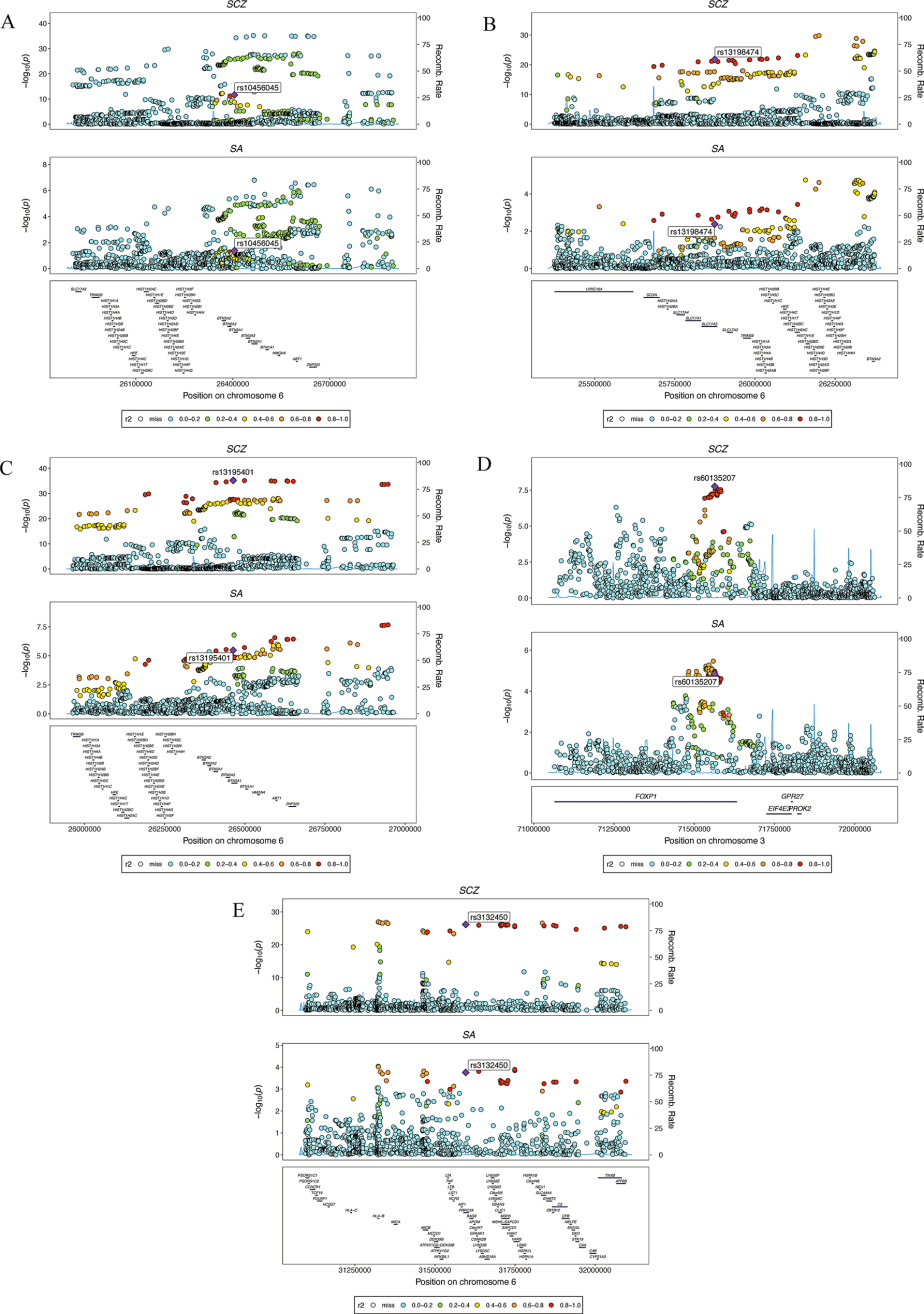
Colocalization analysis of SCZ and SA at newly identified pleiotropic loci is illustrated using stack plots, highlighting a 150 kb Linkage Disequilibrium region surrounding the relevant variants. The X-axis outlines the chromosome position. SNPs are color-coded based on their linkage disequilibrium (r2) with the focal SNP in a European population. (**A**) rs10456045; (**B**) rs13198474; (**C**) rs13195401; (**D**) rs60135207; (**E**) rs3132450. SCZ: Schizophrenia; SA: Suicide attempts

Among annotated SNPs, rs10456045 (PP.H4 97.98%), rs13195401 (PP.H4 93.65%), rs13198474 (PP.H4 95.91%), rs60135207 (PP.H4 89.89%), and rs3132450 (PP.H4 88.68%) showed shared risk loci between the two traits (Table S10) (Fig. 3).

### Mendelian randomization

**Fig. 4:**
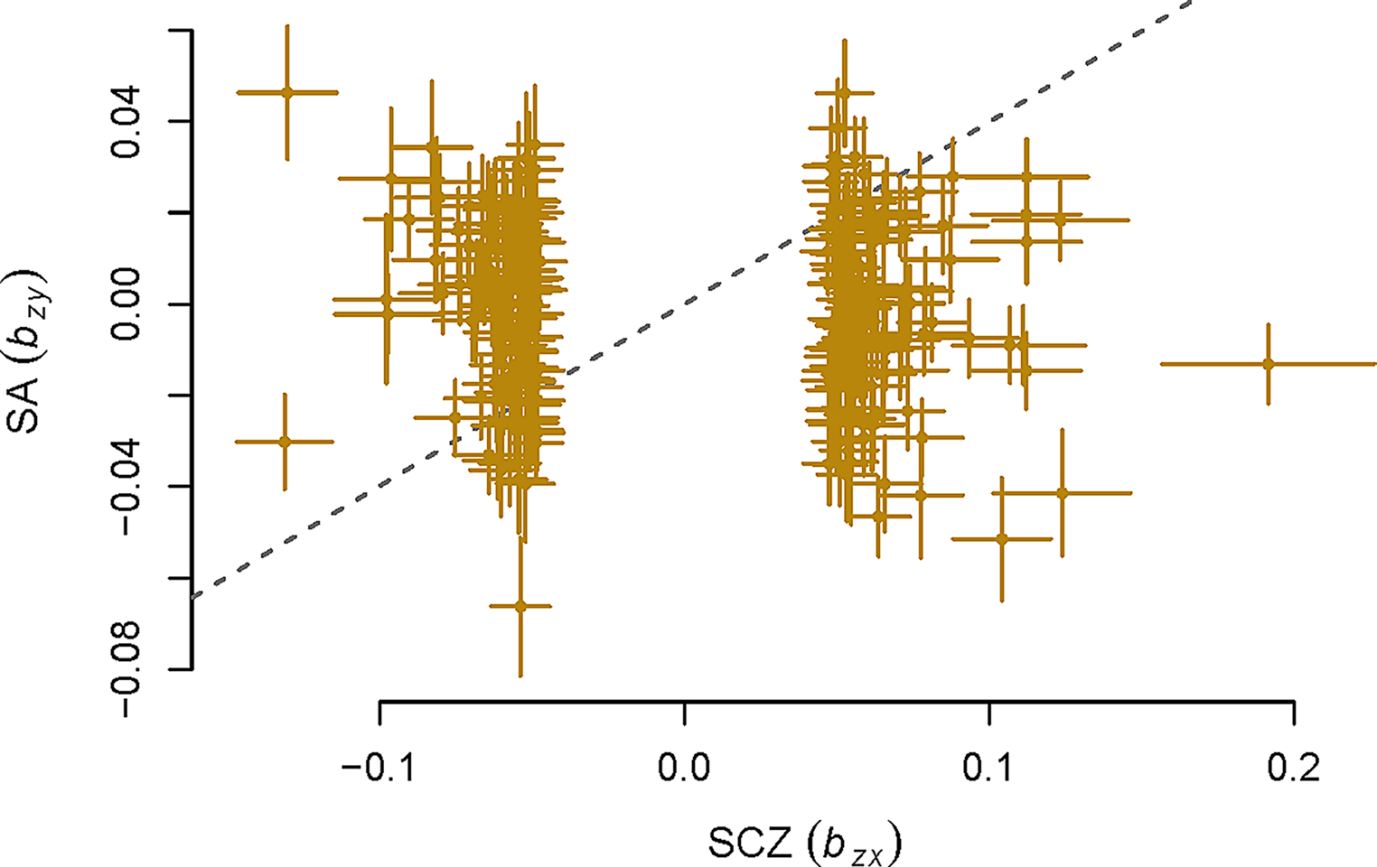
Generalized summary Mendelian randomization (GSMR) analysis of association of SCZ and SA. The estimated effect of the exposure on the outcome, denoted as bxy, is calculated using the ratio bzy/bzx. Bzy is the effect of the SNP instrument on the exposure measured on the logit scale, while bzx refers to its effect on the outcome, isolated from confounding by non-genetic factors. SCZ: Schizophrenia; SA: Suicide attempts

After stringent filtering, 136 IVs were extracted from SCZ for MR analysis, and genetically predicted SCZ was positively associated with the risk of SA (OR 1.17, 95%CI 1.14 to 1.21, *p* = 7.39 × 10^− 22^) according to IVW method. As sensitivity analyses under different circumstances, several complementary MR methods were directionally consistent with primary IVW result, although not all reached statistical difference (such as MR-Egger regression and weighted mode) (Table 2). In reverse MR, genetically predicted SA was not associated with SCZ (*p* = 0.09), indicating the one directional association between the two traits. However, reverse causal result should be interpretated with caution given the result was based on only two IVs after stringent filtration, which limited statistical power and increased potential bias [43]. GSMR also confirmed the positive association between SCZ and SA (beta = 0.40, se = 0.02, *p* = 2.07 × 10^− 58^) (Fig. 4).

**Table 2:** The results of Bidirectional MR and GSMR between SCZ and SA

Exposure-Outcome	Method	SNP	Beta	Se	*P*	OR	OR_lci95	OR_uci95
SCZ-SA	Inverse variance weighted (multiplicative random effects)	136	0.16	0.02	7.39E-22	1.17	1.14	1.21
	MR Egger	136	0.14	0.08	7.87E-02	1.14	0.99	1.33
	Simple mode	136	0.23	0.07	1.91E-03	1.26	1.09	1.46
	Weighted median	136	0.16	0.02	1.20E-14	1.17	1.13	1.22
	Weighted mode	136	0.06	0.07	4.08E-01	1.06	0.92	1.21
	Contamination mixture method	136	0.29	0.02	1.21E-17	1.34	1.3	1.38
	Robust adjusted profile score (RAPS)	136	0.17	0.02	1.63E-19	1.18	1.14	1.23
	Debiased inverse-variance weighted method	136	0.17	0.02	5.70E-22	1.18	1.14	1.22
	Constrained maximum likelihood	136	0.17	0.02	4.70E-26	1.18	1.15	1.22
	Bayesian Weighted Mendelian Randomization	136	0.17	0.02	1.87E-22	1.18	1.14	1.22
	GSMR	229	0.40	0.02	2.07E-58	NA	NA	NA
	MR-CAUSE	148	0.19	0.03	3.92E-03	1.21	1.15	1.28
SA-SCZ	Inverse variance weighted	2	0.21	0.13	9.14E-02	1.24	0.97	1.58
	Contamination mixture method	2	0.21	0.22	1.29E-01	1.23	0.81	1.88
	Robust adjusted profile score (RAPS)	2	0.21	0.14	1.21E-01	1.24	0.95	1.62
	Debiased inverse-variance weighted method	2	0.22	0.13	9.87E-02	1.24	0.96	1.61
	Constrained maximum likelihood	2	0.20	0.13	1.32E-01	1.22	0.94	1.58
	Bayesian Weighted Mendelian Randomization	2	0.22	0.13	1.02E-01	1.24	0.96	1.61
SCZ-SA (outliers corrected)	Inverse variance weighted (multiplicative random effects)	115	0.17	0.01	7.86E-34	1.18	1.15	1.22
	MR Egger	115	0.12	0.06	5.35E-02	1.13	1.01	1.27
	Simple mode	115	0.22	0.06	6.33E-04	1.25	1.1	1.42
	Weighted median	115	0.17	0.02	5.33E-16	1.18	1.13	1.23
	Weighted mode	115	0.04	0.06	5.03E-01	1.04	0.92	1.18
	Contamination mixture method	115	0.26	0.02	2.81E-19	1.3	1.25	1.35
	Robust adjusted profile score (RAPS)	115	0.17	0.02	1.20E-29	1.19	1.15	1.22
	Debiased inverse-variance weighted method	115	0.17	0.01	7.99E-33	1.19	1.15	1.22
	Constrained maximum likelihood	115	0.17	0.02	5.41E-28	1.19	1.15	1.23
	Bayesian Weighted Mendelian Randomization	115	0.17	0.01	8.00E-33	1.19	1.15	1.22

The robustness of MR results was assessed through sensitivity analyses (Table S11). MR-Egger intercept analyses indicated that no horizontal pleiotropy (*p* = 0.73) was observed. The Cochrane Q test confirmed potential heterogeneity (*p* = 1.25 × 10^− 7^). In addition, scatter plots showed a consistent direction of results across methods (Figure S2), funnel plots showed symmetric distribution of IVs (Figure S1). and leave-one-out analysis did not find any outlier SNP (Figure S3). According to the CAUSE method, the negative ΔELPD (-5.298) favored the causal model over the sharing model (Table S12), and the estimated effect remained significant (OR 1.21, 95%CI 1.15 to 1.28, *p* = 0.004) (Table 2 and Figure S4). However, MR-PRESSO and radial MR identified pleiotropy in MR estimates (Figure S5). We therefore eliminated these pleiotropic IVs and re-calculated the MR result. The estimates of the causal effect of SCZ on SA remained positive after outlier removal (OR 1.18, 95%CI 1.15 to 1.22, *p* = 7.86 × 10^− 34^), and no further evidence of heterogeneity (*p* = 0.60) and horizontal pleiotropy was identified (*p* = 0.43) (Tables S11).

### Tissue-level SNP heritability enrichment in SCZ and SA

After Bonferroni correction, we identified SNP heritability enrichment for SCZ across 13 brain-related tissues, led by Brain_Anterior_cingulate_cortex (*p* = 6.87 × 10^− 14^) and Brain_Frontal_Cortex (*p* = 7.60 × 10^− 13^) (Table S14, Figure S6). However, no SNP heritability enrichment for SA was found among 53 tissues after Bonferroni correction (Table S13, Figure S7). Meanwhile, MAGMA gene property analysis was also utilized for tissue-specific identification. As shown in Figure S8, SNP heritability enrichment for SCZ was identified in 14 tissues, led by Brain_Cerebellar_Hemisphere (*p* = 6.56 × 10^− 24^) and Brain_Cerebellum (*p* = 1.05 × 10^− 23^) (Table S16); SNP heritability enrichment SCZ and SA was identified in 10 tissues, led by Brain_Nucleus_accumbens_basal_ganglia (*p* = 3.17 × 10^− 6^) and Brain_Anterior_cingulate_cortex (*p* = 5.54 × 10^− 6^) (Table S15). Of these, SCZ and SA shared 10 brain related tissue enrichments, such as frontal cortex, cerebellum, hippocampus and hypothalamus.

### MRlap analysis

Table S17 presents the MRlap results, which were used to correct for potential bias due to sample overlap, weak instruments, and winner’s curse. The MRlap-corrected estimate remained directionally consistent with the primary IVW result (corrected effect = 0.08, se = 0.01, *p* = 2.18 × 10⁻²⁸). Moreover, the difference between the observed and corrected estimates was negligible (test difference = − 3.81 × 10⁻⁸, *p* = 1.00), suggesting that overlap-related bias was unlikely to have materially affected the inferred causal association between SCZ and SA.

### Gene-level analysis

**Fig. 5:**
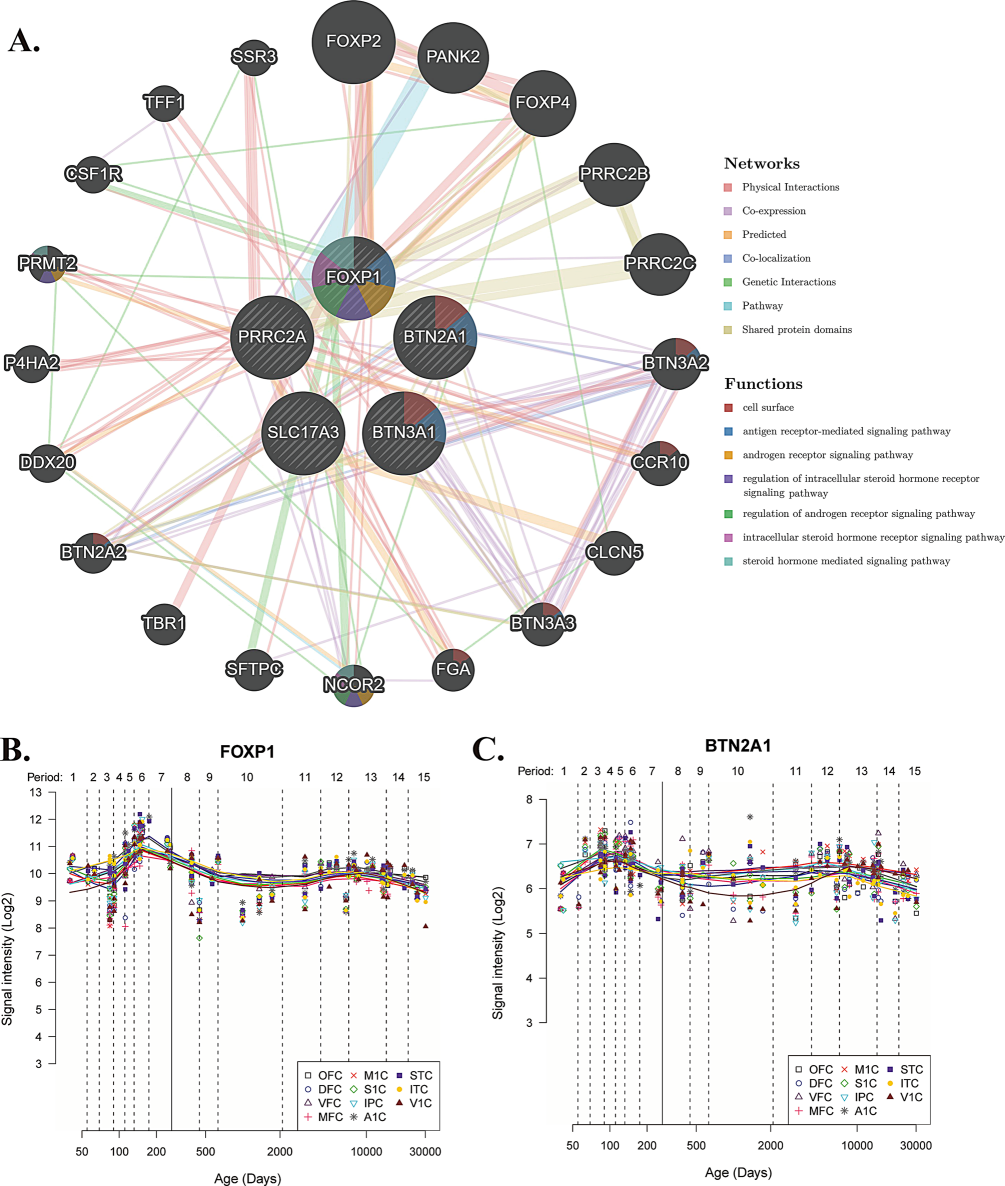

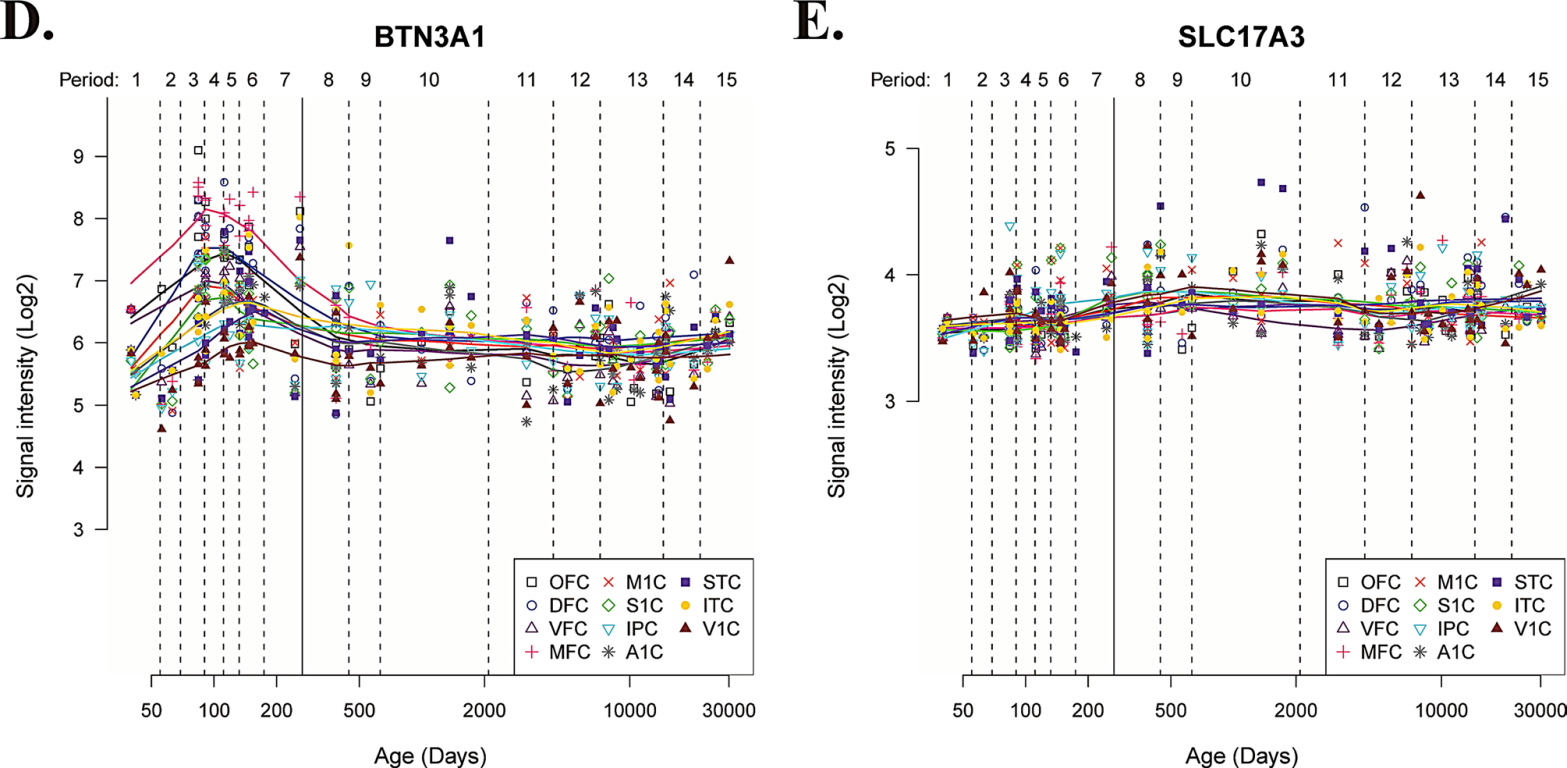
Gene-level analysis of shared genes between SCZ and SA. (**A**) gene-interaction between shared genes and neighboring genes constructed by GeneMANIA; (**B**) Spatiotemporal specific expression profile of FOXP1; (**C**) Spatiotemporal specific expression profile of BTN2A1; (**D**) Spatiotemporal specific expression profile of BTN3A1; (**E**) Spatiotemporal specific expression profile of SLC17A3

GeneMANIA identified 20 frequently mutated genes associated with shared genes, and several biological pathways demonstrated significant association with both traits, including cell surface, antigen receptor signaling pathway, androgen mediated receptor signaling pathway, regulation of intracellular steroid hormone receptor signaling pathway, regulation of androgen receptor signaling pathway, intracellular steroid hormone receptor signaling pathway, and steroid hormone mediated signaling pathway. According to spatial-temporal specific expression analysis, we observed FOXP1 and BTN3A1 showed sharp improvement among different brain regions during infancy period (500 days) and fetus period (80 days), while SLC17A3 showed steady increase with the growing age (Fig. 5).

## Discussion

Previous study merely reported SA is associated with psychiatric disorders with limited genetic liability analysis; this study systematically investigated the common genetic architecture between SCZ and SA by employing multiple and comprehensive genomic statistical methods. Our findings supported the positive genetic correlation between SA and SCZ, identified several genomic regions and shared loci contributing to this overlap, and suggested shared specific enrichment in brain-related tissues, which has not been fully investigated before. MR analyses provided supportive evidence for a potential one-directional causal effect of genetic liability to SCZ on SA, whereas evidence for the reverse direction was limited. Although our findings do not identify an immediately actionable therapeutic target, they provide novel insight for clinical management of comorbidity. Genetic correlation supported the opinion that SA is not solely a psychological complication of SCZ, but may share genetic relationship with SCZ, these findings emphasized earlier risk stratification and closer monitoring of SA in SCZ patients. The enrichment of shared signals in brain tissues and functional annotations pinpointed plausible neurobiological pathways for future target-discovery studies. Colocalization supported further investigation on novel shared variants between phenotypes, providing evidence for identification of risk loci among patients. One-directional causal association supports the clinical importance of optimizing early detection and management of SCZ as part of suicide prevention [44].

Suicide is one of the major public health challenges globally, and the majority of SA occurs in patients with psychiatric symptoms [45]. However, most studies focused on epidemiological and clinical associations between these disorders without much attention on the genetic basis. Mullins et al. Conducted a large-scale GWAS on SA among patients with psychiatric disorders and highlighted the strong correlation between bipolar disorder and SA [46]. As one of the psychiatric disorders that represents a major socioeconomic burden for society, SCZ is considered to be associated with decreased life expectancy and a risk factor for SA [47]. Epidemiological observational studies concluded that 5–10% of patients committed suicide, and 25–35% of them had SA during lifetime [47]. LDSC analysis reveals a positive genetic association between SCZ and SA, which is in accordance with several previous studies that observed a higher incidence of SA in people with SCZ than in the control group [48, 49]. Notably, the observed-scale SNP heritability of SA was substantially lower than that of SCZ. These estimates were derived on the observed-scale heritability and should not be directly compared with the liability-scale heritability reported in the GWAS studies of SCZ and SA because liability-scale estimates are transformed based on disease prevalence and ascertainment rather than case-control proportion [50, 51]. Thus, observed-scale heritability of phenotypes should be interpreted as reflecting differences in common-variant signal strength and phenotype characteristics rather than a direct comparison of diseases’ biological heritability [50].

Based on local genetic correlation between SCZ and SA, 13 independent regions, led by 12q21.31-21.33 (chr12: 23820634–25371083), were identified for SCZ and SA. A recent study conducted by Chang et al. observed 12q21.31-21.32 associated somatic copy number variation (including MGAT4C and NTS) was related to the risk of SCZ [52]. Another region named 6p22.1–p21.33 locates at the extended major histocompatibility complex (MHC), which is one of the most consistently implicated regions in SCZ genetics [53]. Additionally, growing evidence suggested that variation in the MHC/C4 region may be related to SA risk [50]. Except for the representative locus mentioned above, other significant regions also showed positive effects. Consistent direction of local correlation suggests that the shared genetic basis between SCZ and SA is not limited to one isolated locus, but instead reflects a broader polygenic architecture distributed across multiple genomic regions. Partitioned heritability and correlation analysis enriched the interpretation of the shared genetic architecture between them. Rather than being uniformly distributed across the genome, the shared genetic signal appeared to be concentrated among regulatory functional annotations, especially in Human_Promoter_Villar and CpG_Content. This pattern was biologically reasonable considering previous evidence that SCZ risk variants enriched in non-coding regulatory chromatin regions, whereas suicidal behavior has been linked to DNA methylation and broader epigenetic dysregulation [54, 55]. Overall, these findings suggested that the shared liability between SCZ and SA may be mediated through gene-regulatory and epigenetic mechanisms.

Furthermore, we combined significant results from multiple statistical methods and identified 44 novel loci. Colocalization analysis found five SNPs, such as rs13195401 (BTN2A1), rs10456045 (BTN3A1), rs60135207 (FOXP1), rs3132450 (PRRC2A), and rs13198474 (SLC17A3), as shared risk loci between the two traits. BTN3A1, which is abundantly expressed in the cerebral cortex, plays an essential part in the process of antigen presentation (25243493). Tao et al. identified BTN3A1 as a shared genetic variant for both SCZ (38637810), while Li et al. and Jin et al. identified them as potential therapeutic targets for major depressive disorder (MDD) and anxiety [56, 57]. Similar to BTN3A1, BTN2A1 is also correlated to psychiatric disorders, such as MDD, bipolar disorder, and SCZ. Yang et al. reported BTN2A1 demonstrated strong connection between psychiatric disorders and nine brain regions [58]. As a transcriptional repressor, FOXP1 is involved in the maturation of dopaminergic neurons in the midbrain and medium spiny neurons in the striatum, and identified as a high confidence gene for SCZ [59]. According to spatial-temporal specific expression analysis, FOXP1 demonstrated consistent increase among brain regions during infancy period, which supported its’ association with neurodevelopmental disorders, such as autism spectrum disorder, mental retardation, and intellectual disability [59]. Additionally, researchers concluded that FOXP1 contributes to immune responses by driving monocyte differentiation and enhancing lymphocyte proliferation via the NFκB pathway [60, 61], confirming its’ role in the inflammation and immune system involved in SCZ [62]. Compared with other loci, the relevance of SLC17A3 to SCZ or SA is currently less direct. Previous studies have linked SLC17A3 to homocysteine-related variation and ischemic stroke, suggesting that this locus may participate in broader metabolic pathways [63–65]. DNAmocysteine-related genetic variation and altered DNA methylation have also been associated with SCZ in previous studies, providing a plausible indirect link between SLC17A3 and SCZ [66, 67]. However, further studies are required to identify the potential role of these in SA. Therefore, SLC17A3 should be interpreted as potential shared locus that may warrant further functional investigation.

By using TSMR analysis, we observed evidence consistent with a potential causal effect of genetic liability to SCZ on SA. These results are in line with previously reported studies on the causal relationship between SCZ and SA [68]. Cabrera-Mendoza et al. estimated the direct influence of the genetic liabilities on psychiatric disorders and SA through a multivariate MR approach and found that the genetic correlation between them was independent of behavioral traits and psychiatric disturbance [68]. Several potential mechanisms could explain why people with schizophrenia are more likely to be suicidal. The interpersonal theory of suicide suggests that mental disorders increase the risk of social isolation and loneliness, which can lead to despair, discouraging people from seeking and receiving support, increasing their vulnerability to suicide [69]. In addition, genetic variants in the hypothalamic-pituitary-adrenal axis are associated with suicide attempts in patients with schizophrenia [70].

Our results indicate that SNP heritability was enriched in 14 and 10 tissues for SCZ and SA, respectively, with a total of 10 brain tissues for both traits. Analysis of the GTEx dataset revealed concordant tissue-specific genetic enrichment profiles for SCZ and SA, marked by pronounced co-enrichment in cerebral regions. Accumulating evidence supports the notion that SCZ susceptibility spans distributed neural circuitry and is closely linked to structural aberrations across multiple brain areas [71]. Previous research has similarly revealed that SA is linked to structural changes in the brain [72–74], suggesting that potential mechanisms of co-morbidity between SCZ and SA are related to brain structure. Yin et al. compared the subcortical differences between SCZ patients with and without SA, and they observed smaller volumes of the hippocampus and amygdala in SCZ-SA patients [75]. Brain connectivity analysis based on functional magnetic resonance imaging also found increased connectivity in the coupling of the right amygdala and right superior frontal gyrus in SCZ-SA patients [76]. Additionally, Potvin et al. concluded reduced activation of the medial prefrontal cortex in SCZ-SA patients, which was associated with blunted brain reward activity [77]. These findings highlighted the importance of further clarifying the functional relevance of the implicated brain regions.

Several limitations ought to be noted. Firstly, although robust and complement methods were used, one-directional causal interference should be interpreted cautiously considering the influence of residual horizontal pleiotropy, weak instruments, and limited IV number. Secondly, predominant reliance on European population data may restrict the generalizability of findings to non-European ancestry groups. Increasing evidence suggested that the prevalence and clinical manifestations of SCZ vary by race [8, 78]. Therefore, to develop broadly applicable prevention strategies, it is essential to conduct similar analyses across different racial groups. Third, our study provided a correlation between them on a genetic basis; validation of the mechanistic basis underlying the observed genetic associations will necessitate additional clinical investigations and experimental validation in laboratory settings. Fourthly, the relatively low SNP heritability of SA may have reduced statistical power in post genomic analyses, such as genetic correlation, tissue enrichment, and colocalization. Future studies using larger SA datasets will be essential for validating and extending these findings. Finally, the role of environmental factors in the co-occurrence of SCZ and SA needs to be further investigated, given the important role of environmental factors in the etiology of psychiatric disorders.

## Conclusion

To conclude, this study systematically and comprehensively analyzed the genetic correlation and causality between SCZ and SA, unveiling the shared genetic architecture and identifying five novel shared loci between them. These findings provided novel insights into the complicated influence between the two traits, and lay the groundwork for the promising prevention and potential therapeutic strategies for SCZ and SA. Further fundamental and clinical trials are required to validate these discoveries and help better comprehend the underlying mechanisms for the researchers.

## Supplementary Information

Below is the link to the electronic supplementary material.

Supplementary Material 1

Supplementary Material 2

## Data Availability

The authors declare that all enrolled data could be found in the manuscript and supplementary materials. The GWAS summary statistics of SCZ could be found at IEU open GWAS project (https://opengwas.io/datasets/). The GWAS summary statistics of SA could be obtained through submitting application form (https://pgcdataaccess.formstack.com/forms/isgc_data_access_sui).

## Abbreviations

CAUSE: Causal Analysis Using Summary Effect Estimates
CPASSOC: Cross-Phenotype Association
FUMA: Functional Mapping and Annotation of Genome-Wide Association Studies
GSMR: Generalized summary-data-based Mendelian randomization
GTEx: Genotype-Tissue Expression
GWAS: Genome-wide association study
IV: Instrumental variable
IVW: Inverse variance weighted
LD: Linkage disequilibrium
LDSC: Linkage disequilibrium score regression
MAGMA: Multi-marker Analysis of GenoMic Annotation
MR: Mendelian randomization
MR-PRESSO: Mendelian Randomization Pleiotropy RESidual Sum and Outlier
MR-RAPS: Mendelian randomization robust adjusted profile score
MTAG: Multi-trait analysis of GWAS
OR: Odds ratio
PGC: Psychiatric Genomics Consortium
PP.H4: Posterior probability of hypothesis 4
ρ-HESS: Rho-heritability estimation from summary statistics
SA: Suicide attempt
SCZ: Schizophrenia
S-LDSC: Stratified linkage disequilibrium score regression
SNP: Single nucleotide polymorphism
VEP: Variant Effect Predictor
