# Supplementary material for: Deciphering the shared genetic architecture between schizophrenia and suicide attempt: a statistical genomic study
Source: BMC Psychiatry. 2026 Jun 3;26:608. doi: 10.1186/s12888-026-08189-5 (PMC13449581; doi:10.1186/s12888-026-08189-5)
Supplement: Supplementary file 1 [file 12888_2026_8189_MOESM1_ESM.docx]

Figure S1. Funnel plots of the MR estimates. A. MR of SCZ and SA; B MR of SA and SCZ

Figure S2. Scatter plots of the MR estimates. A. MR of SCZ and SA; B MR of SA and SCZ

Figure S3. Leave-one-out plots of the MR estimates. A. MR of SCZ and SA; B MR of SA and SCZ

Figure S4. Scatter plot presents probability of each variant functioning through shared factors and the contribution of each variant to the ELPD test statistic

Figure S5. Radial MR plots identified potential outliers in the MR estimates.

Figure S6 Tissue type-specific enrichment of SNP heritability for SCZ estimated using LDSC-SEG.

Figure **S7** Tissue type-specific enrichment of SNP heritability for SA estimated using LDSC-SEG.

Figure S8 Tissue type-specific enrichment of SNP heritability for SCZ estimated using MAGMA.

Figure S9 Tissue type-specific enrichment of SNP heritability for SA estimated using MAGMA.
